# Exercise Training and *Verbena officinalis* L. Affect Pre-Clinical and Histological Parameters

**DOI:** 10.3390/plants11223115

**Published:** 2022-11-15

**Authors:** Sonia M. Rodrigues Oliveira, Elsa Dias, Ana Paula Girol, Helena Silva, Maria de Lourdes Pereira

**Affiliations:** 1 CICECO-Aveiro Institute of Materials, University of Aveiro, 3810-193 Aveiro, Portugal; 2 Hunter Medical Research Institute, New Lambton Heights, NSW 2305, Australia; 3 Hospital Center of Baixo Vouga, 3810-193 Aveiro, Portugal; 4 Padre Albino University Centre, Catanduva 15806-310, São Paulo, Brazil; 5 Institute of Biosciences, Humanities and Exact Sciences, São Paulo State University (Unesp), São José do Rio Preto 15054-000, São Paulo, Brazil; 6 Department of Biology & CESAM, Centre for Environmental and Marine Studies, University of Aveiro, 3810-193 Aveiro, Portugal; 7 Department of Medical Sciences, University of Aveiro, 3810-193 Aveiro, Portugal

**Keywords:** *Verbena officinalis* L., common vervain, oxidative stress, exercise training, clinical toxicology

## Abstract

*Verbena officinalis* L. or vervain is an herbal medicine and dietary supplement used worldwide. It is used for antidepressant and anticonvulsant purposes, as well as to treat inflammatory disorders, skin burns, abrasions, and gastric diseases, among others. Here, we investigated the biochemical, antioxidant, and histopathological effects of vervain against chronic physical stress. Male Wistar rats were submitted to chronic physical training and oral administration of 200 mg/kg of extract for 7 weeks. Control animals were not treated with either stress or vervain. Body weight was monitored during the study. Liver, kidney, spleen, testis, epididymis, heart, skeletal muscle, and brain samples were collected. Blood cholesterol, lactate dehydrogenase (LDH), bilirubin, and creatinine kinase (CREA), among others, were studied. Glutathione peroxidase (GPox) and superoxide dismutase (SOD) antioxidant activity was analyzed in the blood, liver, and kidney. Testosterone measurements were also performed on whole testis extracts. We found significant weight ratios differences in the epididymis, brain, and heart. Animals submitted to training showed hemorrhagic livers. Kidney histology was affected by both stress and vervain. Cell disruption and vacuolization were observed in the testes and epididymis of animals submitted to stress. Hematological and biochemical markers as CREA, LDH, TP, CKI, URCA, γGT, and glucose revealed statistically significantly differences. Additionally, the activity of glutathione peroxide (GPox) and superoxide dismutase (SOD) in the blood was also impacted. Both stress and vervain have significant in vivo effects. Infusions of vervain include phenylpropanoids, iridoids, verbenalin, hastatoside, and flavonoids, amongst others, which interact synergistically to produce the preclinical effects reported here.

## 1. Introduction

*Verbena officinalis* L. (Verbenaceae), a perennial plant known as vervain, has been extensively used in folk medicine across Europe, West Asia, and North Africa for various ailments such as depression, burns, and inflammatory conditions [1]. The efficacy of the treatment of inflammatory conditions was demonstrated by Speroni and co-workers (2007) [2] in rats orally administered with *V. officinalis* extract. Digestive and antispasmodic properties have also been observed [2]. Furthermore, Calvo (2006) [3] demonstrated the anti-inflammatory and analgesic effect of vervain in rats. Besides, the use of *V. officinalis* as a hemostatic and to treat wounds has been documented by Guarrera and co-workers (2005) [4]. *V. officinalis* also has an extensive record of phytotherapeutic use against respiratory conditions such as catarrh, cough, and bronchial problems [5,6,7,8]. Additionally, Lai and co-workers (2006) [9] showed its neuroprotective effect against the toxicity of the beta amyloid (Aβ) peptide and the reducing agent dithiothreitol in primary cultures of cortical neurons. Anticonvulsant, anxiolytic, and sedative activities have also recently been described, underlying the medicinal application of *V. officinalis* in several neurological disorders, such as epilepsy, anxiety, and insomnia [10,11]. These findings remit to the importance of *V. officinalis* in the prevention of several pathologies, including the prevention of neuronal loss, a characteristic of Alzheimer’s disease. In addition, the infusion of vervain was suggested to inhibit iron availability [1,12], and its essential oil and citral showed pro-apoptotic activity in cancer cell lines, possibly due to the activation of caspase 3 [13], which presents a possible interaction with signaling pathways involved in cancer and apoptosis. The essential oil of vervain consists mainly of isoprenoids, whose preliminary studies have suggested that they have great potential to inhibit the proliferation of tumor cells from human breast adenocarcinoma, leukemia, and colon adenocarcinoma [1,13,14,15]. Moreover, Kou and colleagues (2013) [16] reported the antitumor effect of *Verbena officinalis* L. in H22 tumor-bearing mice and without impaired immune function. A sum of the pharmacological effects of this plant is documented in the British Herbal Pharmacopoeia [17]. Iridoid glycosides, triterpenes, phenylethanol glycosides, and fatty acid esters were also isolated from the common vervain [1,18,19,20]. Moreover, two newly identified secoiridoid glycosides were also extracted from this plant [18]. Antioxidant, radical scavenging, anti-hyperlipemia, and immune-modulatory activities have been associated with secoiridoid glycosides [18,21]. In addition, Duan and colleagues (2011) [22] also described the pharmacokinetics of some flavonoids in a review, where the relationship between some of these compounds and their physiological effect was mentioned. Nevertheless, the exact mechanism of action of *V. officinalis* remains unclear. Moreover, the bio-efficacy and reproducibility of therapeutic effects of herbal compounds are affected by various factors such as the proper identification of plants, season, and collection area; the extraction and purification method; and the concentration and dosage uptake [23,24]. For example, greater levels of phenolic compounds with potent antioxidant abilities were recently reported, when compared to water extracts [25]. Another perspective to increase the antioxidant capacity of *V. officinalis* and to promote its pharmaceutical potential was to use in vitro cultures [26]. Recent research has pointed for the benefits of aqueous extract of *V. officinalis* in traditional practice, since no clastogenic and myelotoxic effect on bone marrow micronucleus of rats was demonstrated, although mutagenic effects against TA98 and TA100 strains were observed in vitro [27]. More recently, Kubica and co-workers (2020) [1] reviewed the medicinal effects of *V. officinalis,* highlighting its role in the food and cosmetics industries. Although there are several studies relating the effects of *V. officinalis* against respiratory affections, and its anti-inflammatory and antidepressant properties, its antioxidant protective potential in vivo against stress remains to be clearly elucidated. Besides cosmetics, an area deeply interested in the application of antioxidants is related to exercise performance. In fact, antioxidant supplementation has received increasing recognition among athletes and professional trainers as a possible viable method for enhancing athletes’ performance [28,29]. As the knowledge on the role of vitamins and phytochemicals and other bioactive compounds amounts, the more the sports industry has been investing the vitamins and antioxidants industry to control anabolic signaling pathways and thus improve the athlete’s stamina and performance. Physical exercise can originate cellular stress, as it increases oxygen consumption and disturbs the intercellular pro-oxidant—antioxidant balance, which triggers the increase in reactive oxygen species (ROS) that react with cellular structures by oxidizing them [30,31]. In addition, moderate exercise acts as a modulator of intracellular antioxidant systems, improving the ROS scavenging activity. Thus, ethnomedicinal supplements are increasingly used by endurance athletes to either after practice recovery or to minimize exercise-induced oxidative stress and ROS [32,33]. Some herbal extracts are then useful in sports, despite that the safety is uncertain [32,34,35,36]. Moreover, since oxidative stress induces cell damage (e.g., DNA inactivation, lipid peroxidation, and denaturation of proteins), it is important to carry out studies to detect changes in the oxidative balance of individuals because of oral intake of health antioxidant foods or beverages, including superoxide dismutase (SOD) of plant origin. Previous studies have shown how supplementation with an antioxidant diet can prevent oxidative damage caused by stressful conditions and after prolonged exercise [37,38,39,40,41]. Vervain has been often suggested to aid muscle strength and recovery while being relatively safe and well-tolerated [42,43]. However, the variances in results are significative and generally illustrate methods that poorly represent human life. *V. officinalis’* antioxidant, anti-inflammatory, neuroprotective, and analgesic properties have been recognized [1,44], and the plant’s broad applications with well-studied bioactive compounds in its composition (e.g., verbascoside, phenolic acids, terpenoids, and isoflavones), along with a common presence worldwide, make it an interesting candidate for biomedical applications in the sports industry and endurance exercise treatments. Therefore, the present study aims to examine the in vivo effects of *V. officinalis* L. on male rats submitted to chronic exercise training. The pro- and antioxidant capacity and the histopathological responses to stress were analyzed on different tissues (blood, liver, kidney, spleen, testes, epididymis, brain, heart, and muscle). As with humans, rat endurance capacity relates closely to liver (and muscle) health. Wistar rats are therefore often used in training protocols and preclinical studies on exercise endurance, as well as for central neural system-mediated responses [45,46,47,48,49].

## 2. Results

### 2.1. Survival, Body Weight, and Organ Ratios

**Figure 1:**
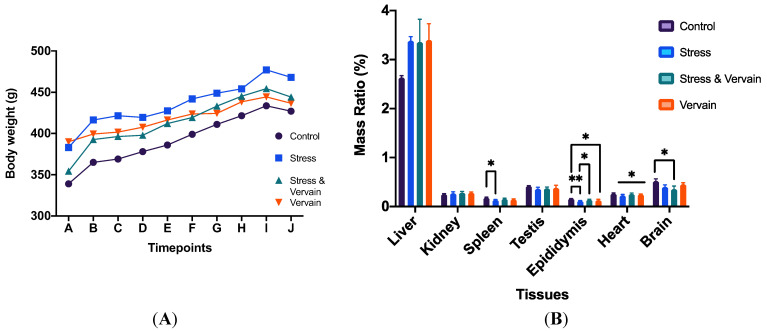
(**A**) Evolution of animal body weight (g)—average mass per group—during the experimental protocol. Letters in the x-axis represent animal handling timepoints. (**B**) Organ/body mass ratio (%) in the several groups. *—Statistically different from control (*p* ≤ 0.05); **—Statistically different from stress group (*p* ≤ 0.01).

In the present study, all animals survived until the end of the research. Animals submitted to stress revealed some signs of fatigue and increased food and water intake compared to those that consumed vervain. The body weight of animals in each group is summarized and illustrated in Figure 1A. No relevant body weight differences were found between the different groups through the protocol. The ratio between the different organs and whole-body weights was calculated, and the results are illustrated in Figure 1B. A statistically significant difference was found in the epididymis/body weight ratio between the control—neither exposed to stress or to vervain—and the other three groups. The stress group was also statistically different from the group treated with stress-plus-vervain. Significant differences between the control group and those submitted to stress and administered with herbal infusions were noted in the brain ratio. On the other hand, no statistical differences were found between groups concerning the liver, kidney, spleen, and testis. Nevertheless, the testis showed slightly higher values of ratio in the control and vervain groups. In addition, the analysis of heart ratio showed significant differences between groups. Nonetheless, the negative control group revealed no significant differences from the positive control group (chronically stressed rats).

### 2.2. Hematological Assays

#### 2.2.1. Blood Smear

**Figure 2:**
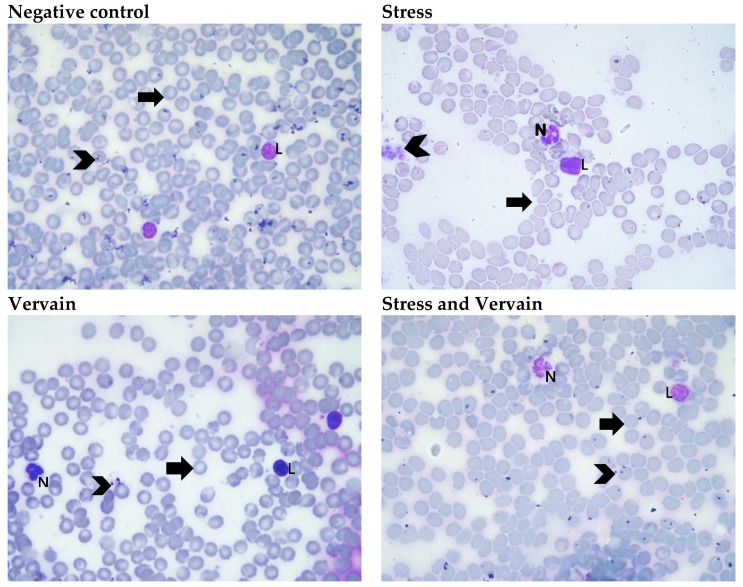
Representative blood smears of animals in each experimental group, Wright’s stain A_t_ = 400×. Arrowhead (platelets), N (neutrophils), L (lymphocytes), Arrow (erythrocytes).

The blood smear presented normal histological patterns in all groups (Figure 2).

#### 2.2.2. Blood Biochemistry

**Figure 3:**
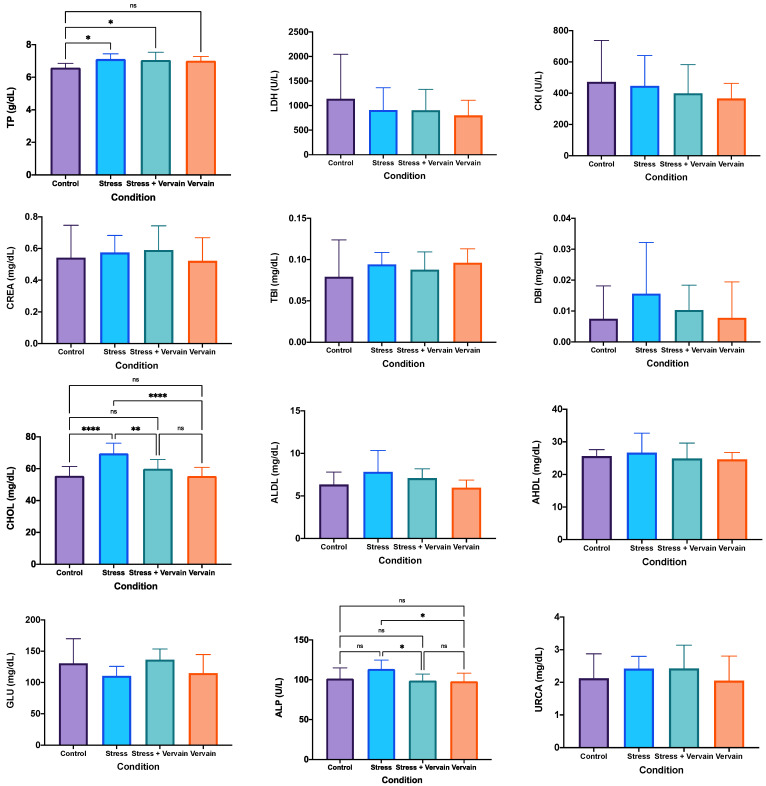

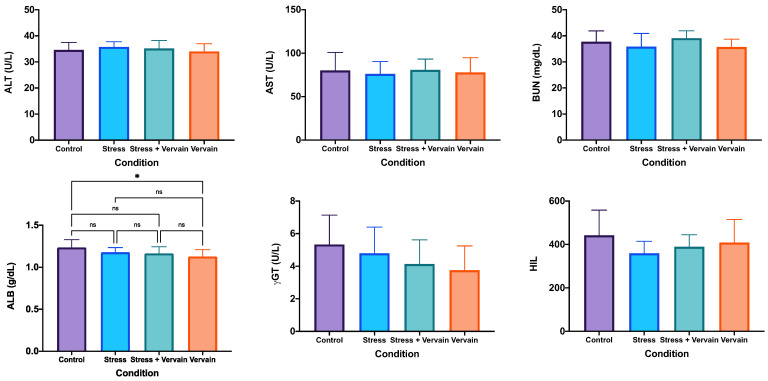
Blood biochemistry results for total protein (TP), lactate dehydrogenase (LDH), creatine kinase (CKI), creatinine (CREA), total bilirubin (TBI), direct bilirubin, aka conjugated bilirubin (DBI), total cholesterol (CHOL), low-density lipoproteins (ALDL), high-density lipoproteins (AHDL), glucose (GLU), alkaline phosphatase (ALP), uric acid (URCA), alanine transaminase (ALT), aspartate aminotransferase (AST), blood urea nitrogen (BUN), total albumin (ALB), and gama-glutamil transferase (γGT), as well as hemolysis, icterus, and lipemia (HIL). Statistically different: *—*p* ≤ 0.05; **—*p* < 0.01; ****—*p* < 0.0001; ns—non-significant.

We screened the animals health by blood testing for different proteins, including total protein (TP), lactate dehydrogenase (LDH), total bilirubin (TBI), direct bilirubin (DBI), creatinine (CREA), cholesterol (CHOL), low-density lipoproteins (ALDL), high-density lipoproteins (AHDL), glucose (GLU), alkaline phosphatase (ALP), uric acid (URCA), alanine transaminase (ALT), aspartate aminotransferase (AST), blood urea nitrogen (BUN), total albumin (ALB), and gama-glutamil transferase (γGT), as well as hemolysis, icterus, and lipemia (HIL)—(Figure 3). The amount of TP obtained in the control group was significantly less compared to the others, namely the values in animals submitted to stress conditions and given vervain (Figure 3). However, no difference was found between the stress and the group submitted to stress-plus-vervain. This suggests the negative effect of stress on the hepatic metabolism, while vervain seem to have been ineffective in fighting these alterations. Nevertheless, no significant changes were detected in the amount of ALT and AST enzymes in the blood, meaning that there is no sign that liver function was severely affected by either stress or vervain. The LDH values in the blood of animals in the control group were higher than those in the vervain group. However, no difference was found between the vervain and the stress group, or the control and the stress-plus-vervain group. Similar results were obtained for creatine kinase (CKI), although a tendency for higher CKI in the negative control group was registered, suggesting that treatments affected inflammation. Similarly, no statistical differences were registered for CREA; however, we noted lower values in animals that just consumed vervain, which may suggest improved kidney function. However, the highest mean value of CREA was observed in the stress-plus-vervain group, followed by the stress group and the negative control. A similar tendency with uric acid (URCA), again suggesting improved kidney function with the consumption of vervain was also registered, which is seemly ineffective against stress effects on kidneys.

Concerning γGT, an enzyme abundantly expressed in kidneys and liver, and of good clinical value for evaluating the hepatobiliary function, its values in the bloodstream decreased with either of the treatments (stress or vervain) (Figure 3).

We also noted some alterations in terms of cholesterol. The group submitted to stress showed higher blood cholesterol levels, indicating changes in the metabolism of this sterol induced by extreme exercise and stress conditions. Animals consuming vervain registered similar levels of blood cholesterol as the negative control. Although no statistical significance was found for ALDL and AHDL, the tendency of the values is similar to that recorded for cholesterol.

We also found statistical difference in the ALP, and enzyme that can screen for liver, gallbladder, or bone health. Particularly noticeable was the decrease of ALP to normal (similar to negative control) levels upon vervain consumption while they were increased in the group submitted just to stress exercise.

Lower values of glucose in the blood collected from the stress group were noted when compared to the other groups. No statistically significant differences were found between groups for values measured in the blood of albumin, ALT, AST, or BUN, leading us not to exclude the variability of random sampling in the observed differences. It is also important to note that some samples had below assay range values that affect the statistical results. HIL was used to assess sample quality and interference with the equipment rather than as an analytical parameter. HIL indices are an objective way to estimate specimen integrity issues that can interfere with laboratory tests (e.g., see [50]).

We also performed hemograms with complete blood counts upon blood testing (Figure S2). We noted that stress induced a decrease of hemoglobin (HGB), mean cell volume (MCV), mean corpuscular hemoglobin (MCH), mean corpuscular hemoglobin concentration (MCHC), cellular or corpuscular hemoglobin (CH), mean platelet volume (MPV), and lymphocytes percentage in the stress animals. Concomitantly, red cell distribution width (RDW), platelet count (PLT), the percentage of monocytes and eosinophils, and the percentage of nucleated red blood cells (NRBCs) increased in the animals submitted to stress conditions. Vervain tisane consumption, by itself, also stimulated the presence of red blood cells (RBCs) and white blood cells (WBCs) and the percentage of neutrophils, monocytes, eosinophils, and of nucleated red blood cells (NRBCs), while decreasing at some level the mean cell volume (MCV), mean corpuscular hemoglobin (MCH), cellular hemoglobin (CH), and mean platelet volume (MPV). Altogether, particularly the factors related with oxygenation, inflammation, and the immune system showed some degree of alterations.

### 2.3. Biochemistry and Antioxidant Status Analysis

#### 2.3.1. Biochemical Status of the Liver and Testosterone Levels in the Testis

**Figure 4:**
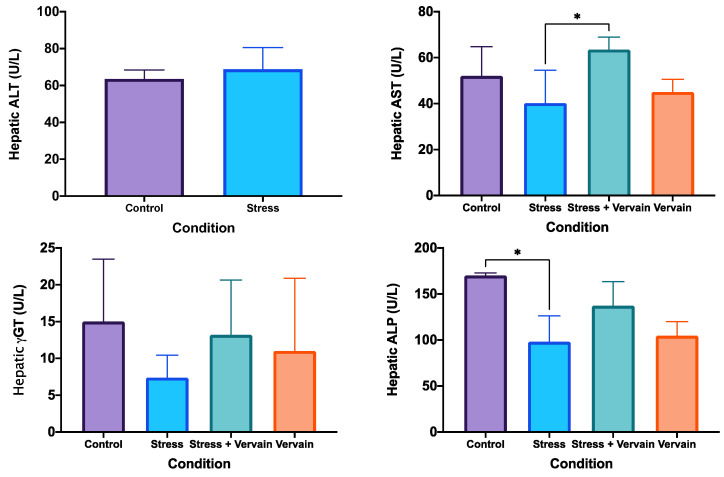
Hepatic enzymes ALT, AST, ALP, and γGT levels in liver homogenates. *—*p* ≤ 0.05.

**Figure 5:**
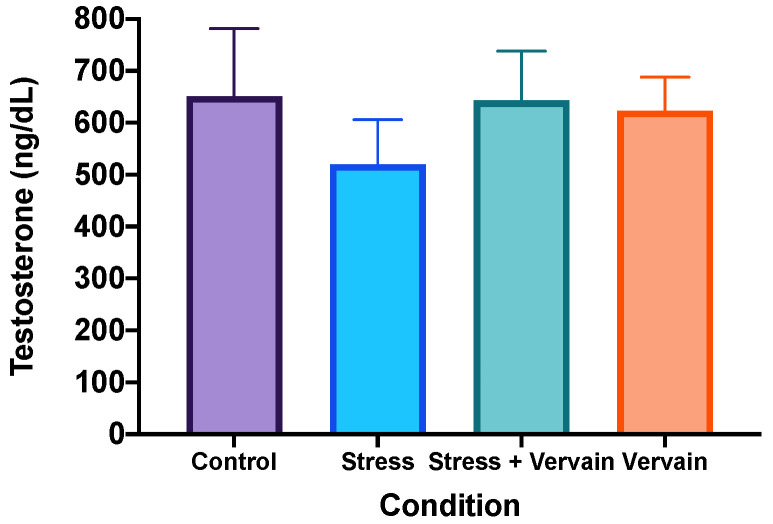
Testosterone levels in testis homogenates.

The analysis of AST, ALT, ALP, and γGT was performed on liver homogenates (Figure 4) and testosterone in testis homogenates (Figure 5). Results of ALP revealed significant differences, particularly between the negative control and the stress groups. Nonetheless, negative control values did not present significant differences in relation to the *V. officinalis* group. Mean values of γGT (Figure 4) and testosterone (Figure 5) were lower in the rats’ organs in the stress group when compared to the other three groups, although no applicable statistical difference was obtained. Rats consuming the herbal infusion showed similar values to the animals in the negative control.

#### 2.3.2. Antioxidant Status of Liver and Kidney

**Figure 6:**
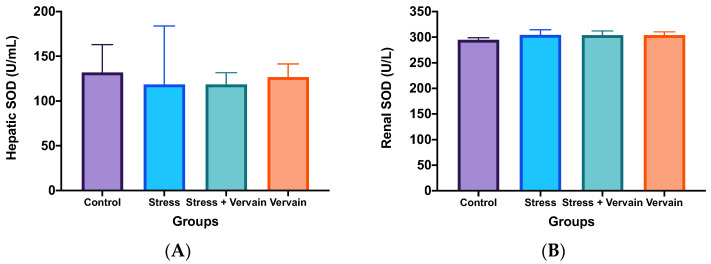
Quantitative determination of superoxide dismutase (SOD) in (**A**) liver and (**B**) kidney. No statistical difference was determined; however, there was more variance in animals submitted to chronic stress, particularly at the hepatic level.

Superoxide dismutase (SOD) and glutathione peroxidase (GPox) activities were investigated in animal tissues (Figure 6) and (Figure S2). For this, the ELISA kits Ransel^®^ and Ransod^®^ were used as per the protocol. Superoxide dismutase (SOD) enzymes are known for catalyzing toxic superoxide anions into hydrogen peroxide and molecular oxygen, clearing the body form the most common free radicals, hence being in the frontline of the defense against reactive oxygen species (ROS)-mediated injury. While glutathione peroxidase (GPox) is a family of enzymes that convert hydrogen peroxide into water, with direct correlation to the levels of selenium, which, in turn, supports immune system and thyroid function while also acting like an antioxidant.

While the body’s primary internal antioxidant defenses, SODs, were altered by both stress and vervain, the statistical significance of these alterations is unclear. Indeed, we observed a tendency for higher SOD activity due to stress in the liver and kidneys (Figure 6), while the results in the blood stream (Figure S2) pointed towards high inter-variability and decreased SOD activity when animals were exposed to both stress and then vervain. Therefore, based on this, we can infer that stress from intensive exercise does affect SOD activity in the body but not that vervain’s tisane antioxidants are significant to stress management and/or oxidative stress homeostasis.

Blood sampling was useful to follow up the body’s antioxidant defenses as the animals adjusted to the different treatment conditions. As oxidative stress and inflammation are very often associated with illnesses, we also investigated the activity of cytosolic selenocysteine compounds like glutathione peroxidase (GPx). GPx acts by scavenging antioxidants, and its analysis can spot selenium deficiency or excess as well as the antioxidant potential of novel drugs or compounds.

GPx displays an inverse correlation with free radicals and consequently oxidative stress (OS). When GPx activity is reduced, antioxidant protection is impaired, resulting in oxidative damage to the membrane fatty acids and functional proteins, resulting in neurotoxic damage. Decreased GPx activity and OS is implicated in the incidence and progression of several health problems, including cardiovascular disease (CVD), diabetes, atherosclerosis, neurodegenerative disorders, and other chronic conditions [51].

**Figure 7:**
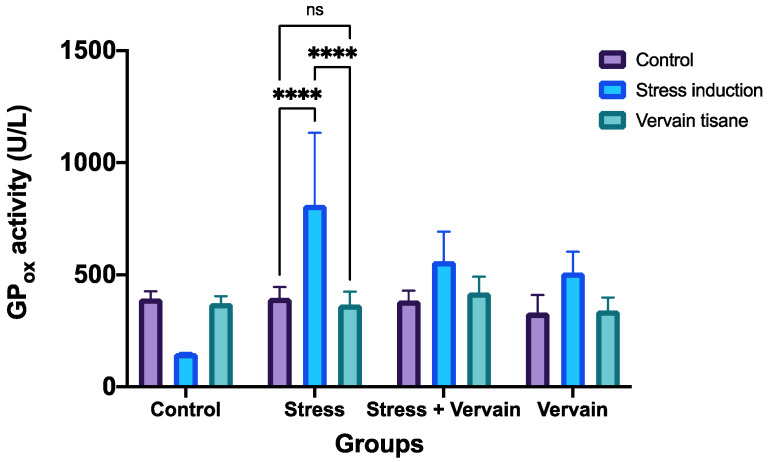
Quantitative determination of glutathione peroxidase (GP_ox_) concentration in whole blood. ****—*p* < 0.0001; ns—non-significant.

Here, at the level of GPox, we observed that stress conditions resulted in a remarkable overexpression of this enzyme that vervain was not able to fully counteract (Figure 7). However, while initial intensive exercise induced high activity of GPx, over time this activity tended to normalize by itself. Vervain tisane appears to aid in this normalization upon consumption. Vervain consumption alone increased GPx levels, which after ~3 weeks of consumption normalized to normal levels. Thus, glutathione peroxidase concentration is affected by both stress or intensive exercise and by vervain tisane consumption, but overtime exposure does not increase or improve antioxidant status as the body works towards homeostasis after ~3 weeks.

### 2.4. Histological Studies

#### 2.4.1. Liver

**Figure 8:**
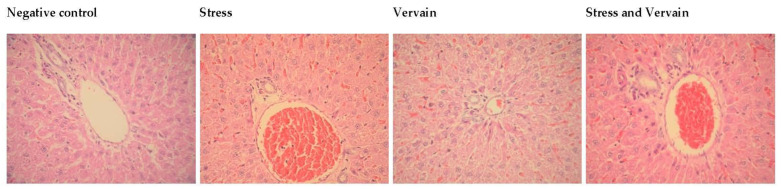
Liver histology under different treatment conditions, H.E. staining, 400×. The first figure represents a normal hepatic structure of animals maintained under normal conditions. The application of stress stimuli to the animals affected the liver histology, particularly via recurrent inflammation and altered hepatocyte structure. The herbal extract was unable to restore normal hepatic morphology in subjects under chronic stress.

Normal hepatic architecture was observed in the negative control rats. In contrast, rats submitted to chronic stress had some inflammation and disarrangements of the hepatic tissue. No significant improvement was observed in the hepatic condition due to administration of the *V. officinalis* infusion. *V. officinalis* alone did not affect the hepatic structure (Figure 8).

#### 2.4.2. Kidney

**Figure 9:**
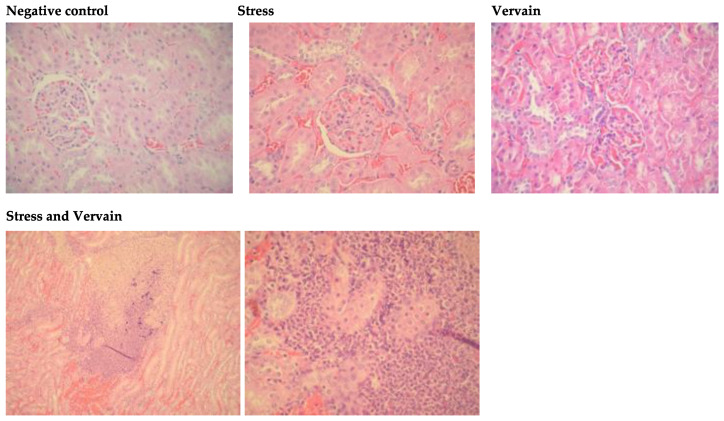
Kidney histology under different treatment conditions, H.E. staining, 400×; bottom line—stress and vervain condition—At = 100× and 400×, respectively. The first figure represents normal renal structure of animals maintained under normal conditions. The application of stress stimuli to the animals affected the kidney histology, with hemorrhages and altered renal structure observed. The herbal extract was unable to restore normal renal morphology in subjects under chronic stress, and the renal cortex and glomeruli were significantly affected by co-exposure to stress and herbal factors.

Renal sections of rats submitted to stress showed some inflammation in the tubules and dilatation of epithelium when compared to negative control group. Severe damage in the renal structure with hemorrhagic areas close to the medulla were observed in the kidneys of rats who were orally administered *V. officinalis*. Additionally, the Bowman’s space was frequently reduced in the glomeruli, and dilation of the tubular epithelium was commonly observed (Figure 9). Due to the filtration function of the kidney, these observations may result from the long exposure to the herbal extract.

#### 2.4.3. Spleen, Brain, and Heart

**Figure 10:**
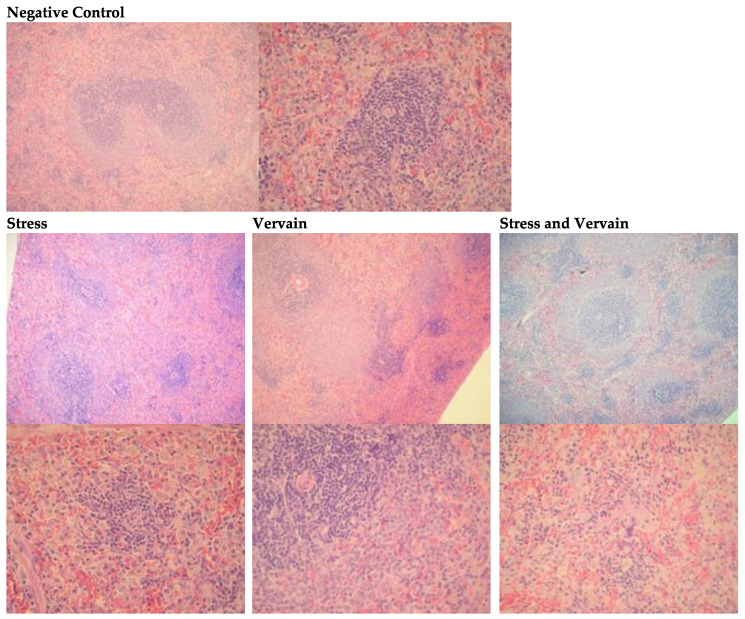
Analysis of histopathologic changes in the spleen, H.E. staining. Two ampliations are depicted: 100× (**top**) and 400× (**bottom**). The first images are representative of the negative controls. No significative changes were observed in the treatment groups; however, giant cells and some iron enrichments (more “brownish” staining) were registered in the animals submitted to acute stress. Splenic organization in the animals submitted to stress factors was affected. These morphological changes were absent in the animals orally administered vervain.

No relevant histological changes were observed in the spleen, brain, and heart of rats in any of the experimental or control groups. Figure 10 shows sections of rat spleen histology.

#### 2.4.4. Testis and Epididymis

**Figure 11:**
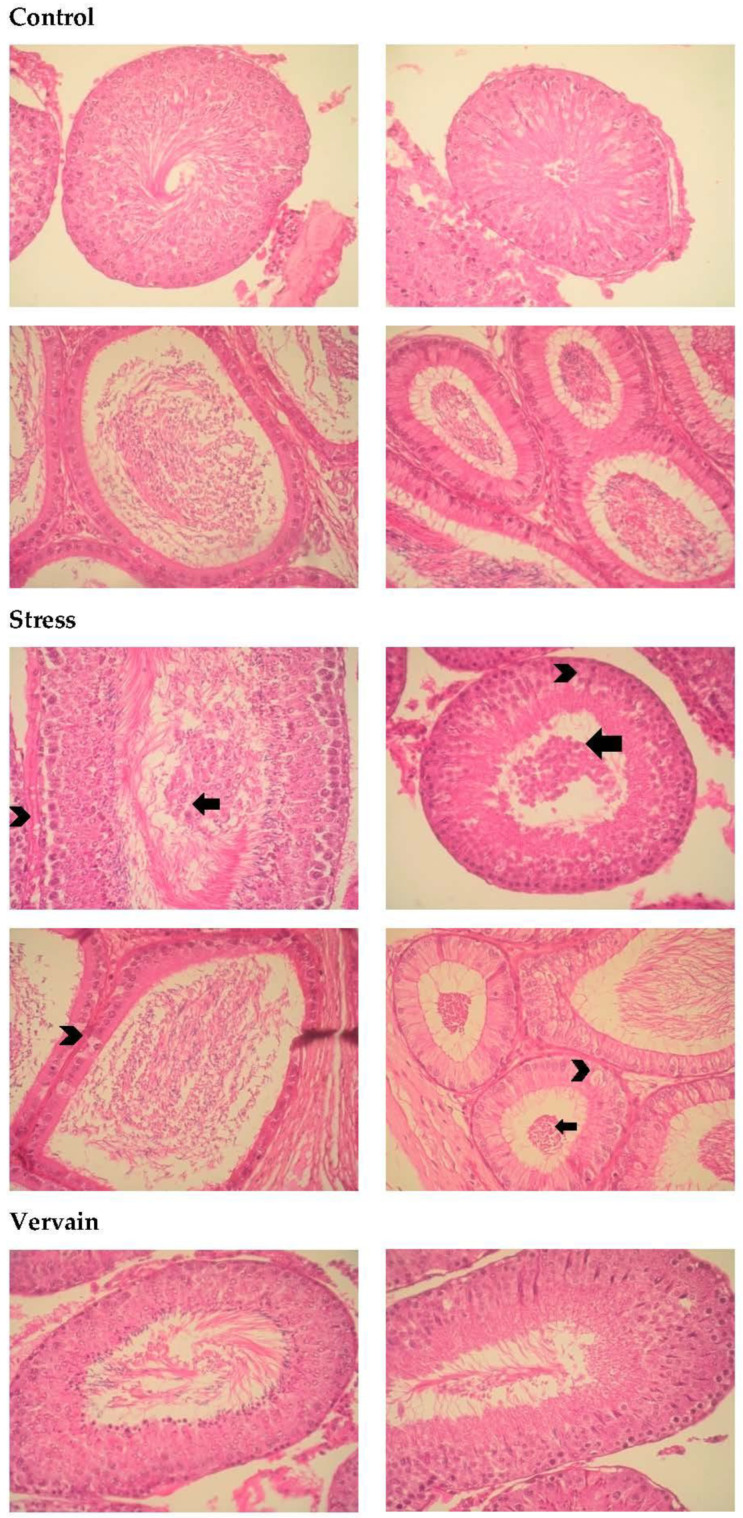

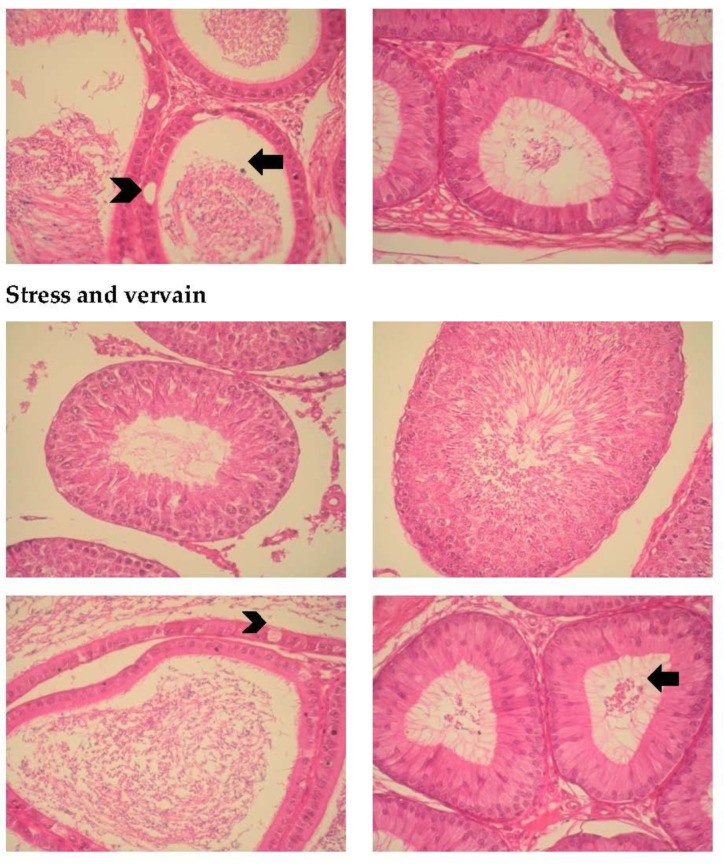
Analysis of histopathologic changes in the testis and epididymis, H.E. staining, 400×. The first images are representative of the negative controls with animals kept under normal conditions. Histological changes were observed in the testis and epididymis of treatment groups. These changes include immature cells (**arrow**) in the lumen of the seminiferous tubules and epididymis and vacuoles (**arrowhead**) in the pseudostratified columnar epithelium of the epididymis.

Significant alterations were observed in testes and epididymis of animals submitted to stress. Figure 11 shows the histology of the testis and “matching” epididymis, respectively, in the different groups. Vacuoles were observed in the epididymis epithelium, showing that vervain herbal extracts can induce significant physiological and/or histological responses in the body. Control and vervain groups showed no major histological changes. Because the effects of the treatment conditions were particularly significant in male reproductive structures, a couple of figures were compiled for each treatment condition as follows: Two testis and epididymis of the same subjects or animals per condition. (A) Negative control, or animals under normal conditions; (B) stress conditioning; (C) oral vervain consumption; and (D) stress followed by vervain consumption.

## 3. Discussion

Intensive exercise and chronic exposure to stress are related to increased generation of reactive oxygen species (ROS) that can lead to the occurrence of several pathologies [27,29,31]. In fact, intensive and continuous overdoing of exercise has been related to different problems, namely heart conditions or damage leading to the development of the designated “athlete’s heart”, which comes as consequence of heart remodeling by intense exercise [27]. The benefits of exercise in the human health are undeniable (reviewed in [52]; however, the understanding of the full dose—response relationship with exercise is incomplete. Moreover, there may be a relationship between intensity and duration of exercise with internal antioxidant capacity, while acute oxidative stress mechanisms following intensive exercise are better understood [53]. After intensive training, the level of antioxidants in the blood and other tissues decreases, while the lipid peroxidation increases. To prevent damage, there is an elaborate antioxidant defense system, which includes enzymatic and non-enzymatic antioxidants, such as uric acid, bilirubin, and albumin. Hence, it is important that the antioxidant defense system in blood, especially in erythrocytes, is effective and recovers properly after exhaustive physical load [36,38,39]. ROS production and, consequently, oxidative stress are higher even after a single event of exhaustive and prolonged exercise [54,55]. To assess oxidative stress and antioxidant status, several biochemical parameters were evaluated in the present study, but only a few revealed significant statistical differences. The importance of biochemistry in medicine is well-established. Clinical biochemistry enables us to easily identify biochemical and physiological changes or associated disorders in the body that can aid in a disease’s diagnosis (see, e.g., [56]). Here, we examined various parameters to assess animals’ health. While many parameters, including complete blood count testing, showed no differences between groups, parameters related to oxygenation, infection, or immune response and inflammation showed some noteworthy differences between control, stress, and vervain conditions.

Parameters, such as HGB, MCV, MCH and MCHC, and MPV (common tests included in a complete blood count), highlighted differences between animals in normal conditions and animals under chronic stress and/or vervain. In the case of intense exercise, because homeostasis is threatened and physiological adaptation systems kick in, it is not surprising that we observed a decrease in HBG values in the stressed animals. In this case, the increased consumption of oxygen from exercising relates to lower HGB values measured, as hemoglobin may be unable to support oxygen uptake during moderate to heavy exercise [57], while RBCs were also stimulated by vervain consumption, suggesting some benefits of vervain consumption ([27]) (Figure S1). Low HGB induced by intensive exercise is compensated with RBC production by the bone marrow, that can also be identified in our blood NRBC data, which report on young (nucleated) RBCs in the blood stream (Figure S1). On the other hand, platelet (PLT) numbers increased while mean platelet volume (MPV) decreased in the stressed animals. When both PLT and MPV are elevated, this suggests bone marrow disorders that decrease the production of platelets. Low MPV may indicate older platelets and that the bone marrow slowed down their production. However, a low MPV with high platelet count suggests infection, or more likely in our case, inflammation [58]. MPV is known to be altered by, for example, altitude, hormones, and intense exercise [59,60]. Therefore, our observations point towards a state of inflammation induced by stress via intensive exercise that was not effectively counteracted by the consumption of *V. officinalis* tisane. Additionally, immune parameters in the complete blood count analysis suggest that while stress is counterproductive for healthy immune conditions, vervain significantly stimulates eosinophil population, and to some extension neutrophils and (at a lesser level) monocytes. So, while moderate exercise is known to improve immune system function, intensive exercise can have the opposite result, as found by other researchers [61,62,63]. A similar relationship can be encountered between exercise and anti-inflammatory responses. While immune function and anti-inflammatory responses are affected, so is the susceptibility to infection. In this situation, although previous reports are scarce on clinical or biochemistry data, *V. officinallis* may indeed have anti-inflammatory and anti-infection, and even antitumoral, properties via increased or improved immune function [1,16,64].

The physiological importance of the liver is well known, and clinical biochemistry allows for an overview of its state [65]. While assessing liver health, no significant changes were identified in the blood levels of ALT and AST enzymes. However, significant statistical differences for the CKI were observed between the control group and the other groups, indicating altered heart function. In fact, mounting evidence suggests that high intensity exercise can effectively increase the risk for heart disorders and cardiac arrest [66]. Significant alterations on uric acid levels and γGT and ALP further pinpoint alterations in liver and kidney health. Glucose is among the main circulating non-enzymatic antioxidant molecules and a scavenger of hydroxyl radicals [40,41,42]. Therefore, the significant decline in serum glucose observed in the animals submitted to stress, namely via exercise, demonstrates a direct vulnerability to extensive damage by hydroxyl radicals, which are the most aggressive member of the ROS family [43]. While the reasons for high total protein values may range from dehydration to cancers like myeloma, they most likely represent elevated levels of inflammation due to experimental conditions.

Antioxidants are molecules that prevent oxidation and can be present as vitamins in food or polyphenols in wine or even enzymes in the body. Glutathione peroxidases and superoxide dismutases are major families ubiquitously expressed as antioxidant enzymes [51,67]. There is mass evidence suggesting that oxidative stress is involved in a wide range of diseases like rheumatoid arthritis, asthma, inflammatory bowel disease, neurodegenerative diseases, diabetes, and even cancer or chronic fatigue syndrome [68].

Antioxidant defenses, like glutathione and catalase, may remove or inactivate the reactive oxygen species [32,38,44]. Additionally, the increase in the antioxidant activity is linearly correlated with the increase in phenolic compounds [45]. Indeed, here we registered increased or altered levels of superoxide dismutase (SOD) and glutathione peroxidase (GPox), both in solid tissues and in blood, and particularly upon exposure to either exercise or herbal extract. The SOD test allows for research into the effects of sports/stress on free radical damage and the protective effects of SOD (e.g., [69]).

Our blood biochemistry analysis pointed towards increased inflammation due to intensive exercise stress. Indeed, oxidative stress imbalance and inflammation are often associated with most human diseases (e.g., [70,71]), and intensive exercise, associated with dysfunctional oxidation, can also be associated with a number of disorders [31,46,72]. Our antioxidant studies further confirmed this altered oxidative balance along with some inter-variability amongst the animals. Altogether, our results suggest the working of pathways or mechanisms that worked to re-establish the redox balance of the body particularly after longer periods of exposure. Autonomic regulation and epigenetic mechanisms can justify some of the interplay observed between exercise, oxidative stress, blood biochemistry, and histopathological effects [73]. Concomitantly, vervain tisane positively affects biochemical and antioxidant status at a non-statistically significant level. Histology analysis also confirmed the effects of stress on the tissues as well as some unexpected reproductive effects that were further highlighted by investigating testosterone concentration in testis homogenates. The role of gonadal steroids in the regulation of systemic antioxidants is not known, nor is whether the compounds of *V. officinalis* L. extracts affect these steroids or antioxidants. Previous studies have related herbal extracts to reproductive toxicology [47,48,49,50]. These herbal preparations should therefore be better studied, regardless their beneficial effects previously reported on other organs. The mechanisms by which the observed effects are caused remain unclear, particularly at the histological level. Looking at other herbal preparations, Singh and Singh (2009) [51] found no evidence of the Brahmi (*Bacopa monnieri*) extract to interfere with the secretion of testosterone by Leydig cells. Thus, they postulated that a direct action on the seminiferous tubules could occur. Vacuoles have been reported to occur in Sertoli cells of these tubules in rat testis after being treated with gossypol [52]. Other extracts, such as those of ginseng or Tribulus [74] or *Sophora pachycarpa* [75,76] (also used as antioxidants), have been reported to improve male reproductive status, while others, such as those of *Tropaeolum majus* L. leaves, have bene shown to be toxic for male meiosis and testicular and seminiferous tubules morphology [77]. Therefore, given our results, we cannot exclude the hypothesis of the *V. officinalis* infusion causing suppression of spermatogenesis through the Sertoli cells. Administration doses, time course, and reversible alterations after cessation of the treatment should also be considered. Numerous investigations applying different techniques or methods, such as HPLC to aqueous extracts of *V. officinalis* (inclusively in the Iberian Peninsula), reported that the main components of a vervain infusion ought to belong to the classes of iridoids, phenylpropanoids, flavonoids, luteolin, and terpenoids [12,20,53,54,55]. Most if not all these components have shown significant antioxidant activity in in vitro models, particularly related to phenolic and flavonoid contents, but also to the verbascoside radical scavenging compounds found in many members of the *Verbenaceae* family [56]. In fact, aqueous extracts from *V. officinalis* have been previously characterized and reported significant numbers of iridoids, flavonoids, and phenolic acids [78]. Indeed, vervain represents a good source of these antioxidants and of other mineral compounds and trace elements important for human health [78]. Iridoids are demonstrated to have hepatoprotective, anti-inflammatory, hypoglycemic, hypolipidemic, antitumor, and neuroprotective properties [79], while flavonoids have been reported to aid testicular steroidogenesis and general testicular function via antioxidant pathways [80,81]. Phenolic acids or polyphenols are maybe the most popular and well-studied bioactive compounds with significant antioxidant (and more recently, pro-oxidant) properties registered in the literature [82,83]. The widespread availability and accessibility of polyphenols as nutritional supplements and fortified foods may represent a hazard for reproductive health upon excessive consumption. It is reported that these molecules can regulate key targets of oxidative stress and inflammation that are also involved in fertility and development [84,85,86]. Moreover, most herbal compounds can act in synergism potentiating a panoply of preclinical effects in animal metabolism. Polyphenols, for example, can neutralize free radicals and improve fertilization rates by protecting germ cells and eggs from oxidative stress, while they can also lead to hormonal imbalance and reduced fertility upon the presence of isoflavones metabolites [87]. Here, while we observed important alterations at both biochemical and morphological level induced by stress and/or intensive exercise, the aqueous extract of vervain did not restore morphological parameters or biochemical and oxidative markers and showed interesting results in the testis and epididymis histology. Although we tried to mimic a dosage at which a human would consume a commercial vervain tisane, dosage and duration affect physiology in a non-linear way. These synergistic effects can involve different mechanisms as a compound or mixture of compounds that affect a single target or tissue, or, for example, that can antagonize or counteract the development of cancer or drug resistance [46,57,58]. Therefore, *V. officinalis* L. can hold a new source of bioavailable compounds and functional foods of nutraceutical importance for animal and/or human health, for example, as an anti-inflammatory [59]. Nonetheless, careful considerations on herbal bioactive substances dosage and source must be detailed before advancing to the trial phases. Thus, strong collaborations are important for novel comprehensive studies that include real-life human conditions. Paradoxically, and despite the decreased investment in ethnopharmacological research, the literature supports the ethnomedicinal advantages of plants, with the main disadvantage probably being the fast metabolism and/or poor characterization in vivo of phytochemical formulations. Moreover, depending on the compounds, it may be necessary to use the application of novel delivery methods to achieve the intended outcome, thus avoiding overdoses or unknown metabolism factors. Further investigations are warranted and ongoing to determine the mechanisms involved in the data reported here. Nonetheless, our results highlight that vervain consumption (as a dietary supplement for athletes) against intensive exercise is not enough for recovery or to prevent oxidative stress, and it can have undesirable effects in different tissues.

## 4. Materials and Methods

### 4.1. Plant Material

Specimens of *Verbena officinalis* L. were collected from the Campus of Santiago, University of Aveiro, Portugal; coordinates: 40°38′00 N 8°39′35 W (^®^Google Earth). A voucher specimen was identified by the botanist Prof. Helena Silva and deposited in the Herbarium of the Department of Biology, University of Aveiro, Portugal (AVE), under the reference number AVE5494. Air-dried samples (10g) were ground and infused in boiled water (1 L). This infusion was then filtered, frozen at −80 °C, lyophilized (Lyophilazer Snijders Scientific, type 2040) for 48 h, and kept in a humidity-free atmosphere until further use.

### 4.2. Animals and Experimental Protocol

Twenty male Wistar rats (315–435 g) purchased from Harlan (Barcelona, Spain) were housed in standard laboratory conditions of temperature (23 ± 2 °C), humidity 60 ± 5%, and natural 12 h light/dark cycle with free access to the standard pellet chow and drinking water ad libitum. Animals were randomly divided into 4 groups, (n = 5/group): (1) a training group (daily physical training and untreated); (2) a stress-plus-vervain treatment group; (3) vervain treatment group (not subjected to training); and (4) negative control group (not stressed or treated).

**Figure 12:**
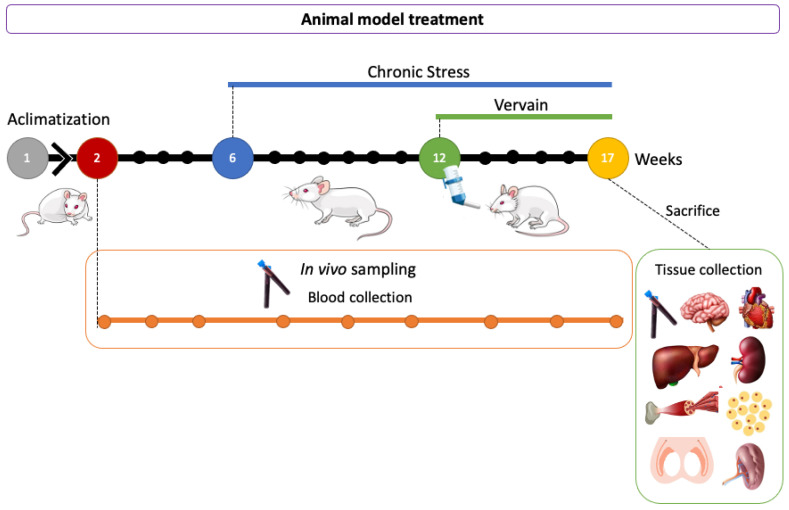
Temporal diagram representation of the in vivo protocol. Animals were acclimatized to the local conditions for 7 days. Then, three blood samples per animal were collected for each experimental condition: under no training induction or herbal gavage, submitted to chronic stress, and finally when orally given vervain (200 mg/kg) tisane. Animals were left to recover for some days (12 days) between each blood collection. Vervain was administered for about a month to evaluate longer exposure effects. Finally, all the animals were sacrificed. In total, nine blood samples per animal were collected, with three per experimental condition (normal, under stress, and under stress with vervain consumption). Several solid tissues were also collected for further analysis as follows: liver, kidney, spleen, testis, epididymis, heart, brain, skeletal muscle, and fat tissue (white adipose tissue).

Animal trials were conducted in agreement with the national guidelines and the European Directive (2010/63/EU) for ethics in animal experiments and in compliance with the commonly accepted ‘3Rs’. European Community guidelines (EEC Directive of 1986; 86/609/EEC) for the care and use of animals were followed. MdLP holds special animal use certification from the Portuguese Competent Authority, the Direção Geral de Veterinária (in accordance with the num 3 of Portaria number 1005/92 from October 23rd). Initially, three blood samples were taken from all rats in all groups. Then, rats from groups 1 and 2 were submitted to daily physical stress as previously described by Cerqueira et al. (2007) [37]. Briefly, animals submitted to stress were exposed to random stimuli (overpopulation, cold water, hot air, noise, and vibration) daily for at least 30 min per day, and at random times of the day to avoid habituation. Blood samples were collected from the tail every 12 days into heparinized tubes for further analysis. After 12 days from the onset of stress induction, another three blood samples were taken and the treatment with vervain extract was started for groups 2 (stress-plus-vervain) and 3 (vervain only). The lyophilized extract was dissolved in water and administered to the animals orally (200 mg/kg body weight) for 55 days. During this period, three blood collections followed to monitor the effect of the herbal treatment. The weight of animals was monitored through the experiments. At the end of the stress and treatment protocols, animals were anesthetized and euthanized. Liver, kidneys, spleen, and heart, as well as samples of skeletal muscle, brain, testis, and epididymis, were collected and weighted. Some samples of these organs were prepared for histology, and other fragments were stored at −80 °C for further analysis. A diagram representing the in vivo protocol is represented in Figure 12.

### 4.3. Blood Hematology and Biochemistry Assays

Blood smears from all groups of animals were stained by Wright’s stain and observed under a light microscope Olympus BX41 coupled with to a digital camera. Hemograms were also performed (ADVIA 2120, Siemens). The plasma of the remaining blood was separated by centrifugation at 15,000× *g* for 10 min. Then, a panel of biochemical analysis (Dimension RXL, Siemens) for hepatic, renal, and cardiovascular markers of damage, regeneration, and general health was studied including: alanine aminotransferase (ALT), aspartate aminotransferase (AST), direct bilirubin (DBI), total bilirubin (TBI), total protein (TP), uric acid (URCA), lactate dehydrogenase (LDH), creatinine (CREA), chronic kidney insufficiency creatinine kinase (CKI), glucose (GLU), gamma-glutamyl transpeptidase (γGT), high-density lipoprotein (HDL), total cholesterol (CHOL), low-density lipoprotein (LDL), alkaline phosphatase (ALP), blood urea nitrogen (BUN), and albumin (ALB).

#### Antioxidant Status Investigation

The activity of glutathione peroxidase (GPox) and superoxide dismutase (SOD) in the blood was assayed spectrophotometrically using commercial Ransod and Ransel kits purchased from Randox Laboratories Ltd. (Portugal) on an Olympus AU 2700 machine. The kits were used as previously described by Faix et al. (2007) [38] and following the recommendations of the fabricant. For SOD determinations, erythrocytes were isolated from whole blood immediately after the blood collection by centrifugation at 2500 rpm for 10 min, at room temperature. Then, they were washed three times in 0.9% NaCl solution, centrifuged for 5 min at 3000 rpm between washings, and stored at −80 °C until analysis. For GPox activity assays, 130 µL of fresh whole blood was collected and stored at −80 °C until analysis. Samples were hemolyzed with ice cold distilled water (1/5), cell membranes removed by centrifugation, and the supernatant was used for analysis.

### 4.4. Liver and Kidney Analysis

#### 4.4.1. GST Activity

Samples of liver and kidneys from all groups were used to evaluate the activity of glutathione-S-transferases (GSTs). After being homogenized in 1ml of phosphate buffer of 50 mM (pH = 7.0) with 0.1% Triton X-100 and centrifuged at 15,000× *g* at 4 °C for 10 min, the activity of GSTs was determined by the formation of a GSH conjugate with the substrate 1-chloro-2,4-dinitrobenzene (CDNB), and it was spectrophotometrically measured at 340 nm. Results are expressed as nmol/min/mg protein (mU/mg).

#### 4.4.2. Total Protein

The total protein content of liver and kidney homogenates was evaluated spectrophotometrically at 594 nm using Bradford Reagent (Applichem^®^, Darmstadt, Germany) and bovine serum albumin (BSA) as standard. The protein content was quantified to normalize the results obtained for the enzymatic activities.

### 4.5. Histological Studies

Samples of liver, right kidney, spleen, heart, and brain were fixed in 4% formaldehyde, dehydrated in series of graded ethanol, cleared in benzene, and embedded in paraffin. Sections of 2–4 µm thickness were stained with hematoxylin-eosin (H.E.). Right testis and epididymis from all rats were fixed in Bouin’s fluid and then prepared for the usual histological procedure. All sections were observed using an Olympus BX41 light microscope coupled to a photographic system.

### 4.6. Statistical Analysis

Data were expressed as mean ± S.D and treated by analysis of variances for differences between groups using GraphPad Prism 6 software. Differences between groups were considered statistically significant if *p* ≤ 0.05.

## 5. Conclusions

The mass ratio of some organs (epididymis, brain, testis, and heart) was calculated being statically different between groups. Some blood biochemical markers (CREA, LDH, TP, CKI, URCA, ΥGT, and glucose) showed statistical differences amongst the groups and signalized cardiac, hepatic, and renal health alterations. The blood smear and hemogram were normal. Histology of testis and epididymis was performed, and some changes were observed significantly in the testis. Changes in non-enzymatic components of the glutathione system in whole blood and RBCs highlighted different redox conditions. This can signal alterations in the antioxidant mechanisms that altered RBCs membranes as some literature describes. Our study showed significant effects on health induced by physical stress or intensive exercise and by vervain, *V. officinalis*, aqueous extract. The results of this investigation are relevant from the point of view of the consumption of dietary supplements in athletes. Further studies are ongoing to better understand the effects of *V. officinalis* L. at pre-clinical levels.

## Supplementary Materials

The following supporting information can be downloaded at: https://www.mdpi.com/article/10.3390/plants11223115/s1, Figure S1: Hemogram results from the different treatment conditions. WBCs, white blood cells; RBCs, red blood cells; HGB, hemoglobin; HCT, hematocrit; MCV, mean corpuscular volume (RBC average size); MCH, mean corpuscular hemoglobin; MCHC, mean corpuscular hemoglobin concentration; CHCM, cellular hemoglobin concentration mean; CH, cellular hemoglobin; RDW, red cell distribution width; HDW, hemoglobin distribution width; PLT, platelets; MPV, mean platelet volume; NEUT, neutrophils; LYMPH, lymphocytes; MONO; monocytes; EOS, eosinophils; BASO, basophils; LUC, large unstained cells; and NRBC, nucleated red blood cells. *—*p* ≤ 0.05; **—*p* < 0.01; ***—*p* < 0.001; *p* < 0.0001; ns, non-significant. $\bar{x}$, mean value; Figure S2: Superoxide dismutase, SOD, activity (Randox^®^) in whole blood of rats. No significant differences in SOD activity were registered due to stress or vervain administration. Median marked with horizontal bar. ns, non-significant *p* value; Table S1: Hemogram reference values. Values primarily based on veterinary Hematology: Oscar Schalm Publ. Bailliere, Tindall & Cassell Ltd., London, 1965, p. 51. References to Coulter^®^ pages 102–105, same volume. All Units SI units. From ^®^ Coulter Electronics Limited, Coldharbour Lane, Harpenden, Herts.
