# Supplementary material for: Exercise Training and Verbena officinalis L. Affect Pre-Clinical and Histological Parameters
Source: Plants (Basel). 2022 Nov 15;11(22):3115. doi: 10.3390/plants11223115 (PMC9699298; doi:10.3390/plants11223115)
Supplement: Supplementary file 1 [file plants-11-03115-s001.zip › plants-1920331-supplementary.pdf]

plants

MDPI

Article

Exercise training and *Verbena officinalis* L. affect pre-clinical and histological parameters

Sonia M. Rodrigues Oliveira[1,2*], Elsa Dias[1,3], Ana Paula Girol[4,5], Helena Silva[6], Maria de Lourdes Pereira[1,7*]

[1] CICECO-Aveiro Institute of Materials, University of Aveiro, 3810-193 Aveiro, Portugal
[2] Hunter Medical Research Institute, New Lambton Heights, NSW 2305, Australia
[3] Hospital Center of Baixo Vouga, 3810-193 Aveiro, Portugal
[4] Padre Albino University Centre, Catanduva, São Paulo, 15806-310, Brazil
[5] Institute of Biosciences, Humanities and Exact Sciences, São Paulo State University (Unesp), São José do Rio Preto, São Paulo, 15054-000 Brazil
[6] Department of Biology & CESAM, Centre for Environmental and Marine Studies, University of Aveiro, 3810-193 Aveiro, Portugal
[7] Department of Medical Sciences, University of Aveiro, 3810-193 Aveiro, Portugal
* Correspondence: sonia.oliveira@ua.pt (S.M.R.O.); mlourdespereira@ua.pt (M.d.L.P.)

Abstract: *Verbena officinalis* L. or vervain is an herbal medicine and dietary supplement used worldwide. It is used for antidepressant and anticonvulsant purposes, as well as to treat inflammatory disorders, skin burns, abrasions, and gastric diseases, among others. Here, we investigated the biochemical, antioxidant, and histopathological effects of vervain against chronic physical stress. Male Wistar rats were submitted to chronic physical training and oral administration of 200 mg/kg of extract for 7 weeks. Control animals were not treated with either stress or vervain. Body weight was monitored during the study. Liver, kidney, spleen, testis, epididymis, heart, skeletal muscle, and brain samples were collected. Blood cholesterol, lactate dehydrogenase (LDH), bilirubin, and creatinine kinase (CREA), among others, were studied. Glutathione peroxidase (GPox) and superoxide dismutase (SOD) antioxidant activity was analyzed in the blood, liver, and kidney. Testosterone measurements were also performed on whole testis extracts. We found significant weight ratios differences in the epididymis, brain, and heart. Animals submitted to training showed hemorrhagic livers. Kidney histology was affected by both stress and vervain. Cell disruption and vacuolization were observed in the testes and epididymis of animals submitted to stress. Hematological and biochemical markers as CREA, LDH, TP, CKI, URCA, γGT, and glucose revealed statistically significantly differences. Additionally, the activity of glutathione peroxide (GPox) and superoxide dismutase (SOD) in the blood was also impacted. Both stress and vervain have significant in vivo effects. Infusions of vervain include phenylpropanoids, iridoids, verbenalin, hastatoside, and flavonoids, amongst others, which interact synergistically to produce the preclinical effects reported here.

Keywords: *Verbena officinalis* L.; common vervain; oxidative stress; exercise training; clinical toxicology

Supplementary Materials:

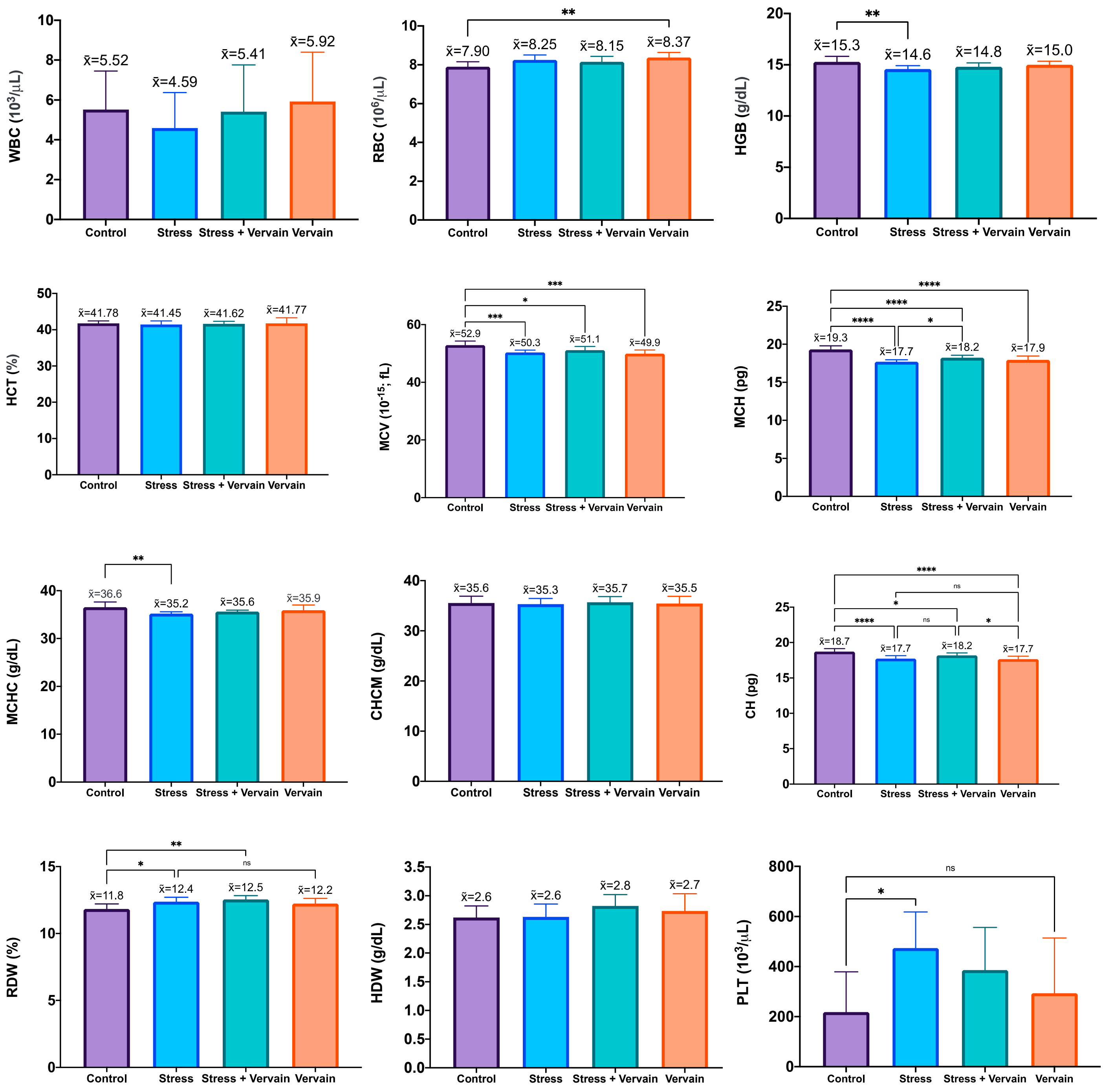

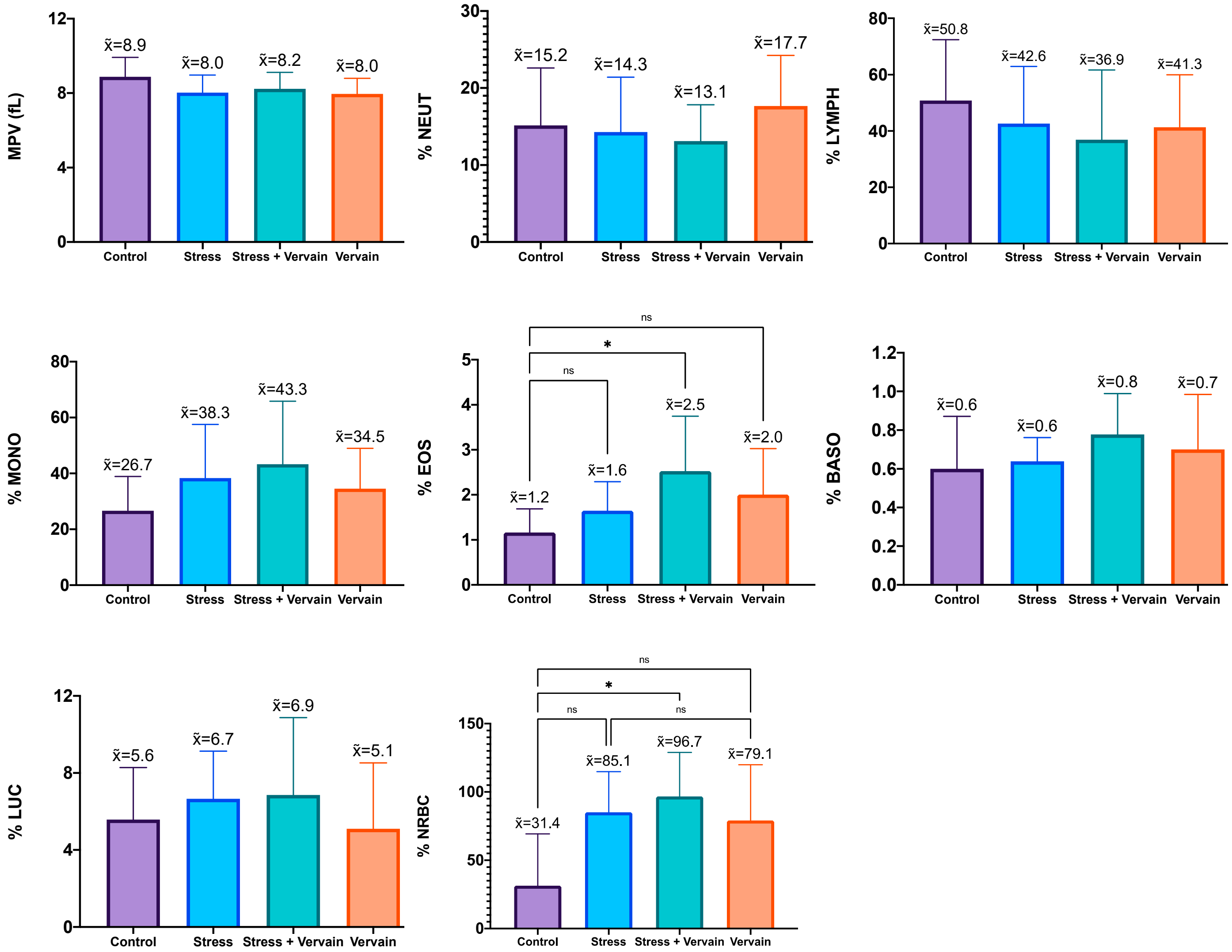

Figure S1. Hemogram results from the different treatment conditions. WBC, white blood cells; RBC, red blood cells; HGB, hemoglobin; HCT, hematocrit; MCV, mean corpuscular volume (RBC average size); MCH, mean corpuscular hemoglobin; MCHC, mean corpuscular hemoglobin concentration; CHCM, cellular hemoglobin concentration mean; CH, cellular hemoglobin; RDW, red cell distribution width; HDW, hemoglobin distribution width; PLT, platelets; MPV, mean platelet volume; NEUT, neutrophils; LYMPH, lymphocytes; MONO; monocytes; EOS, eosinophils; BASO, basophils; LUC, large unstained cells; NRBC, nucleated red blood cells. * - $p \leq 0.05$; ** - $p<0.01$; *** - $p<0.001$; **** - $p<0.0001$; ns - non-significant; $\tilde{x}$ - mean value.

Table S1. Hemogram reference values. Values primarily based on veterinary Hematology: Oscar Schalm Publ. Bailliere, Tindall & Cassell Ltd., London, 1965, p. 51. References to Coulter® pages 102-105, same volume. All Units SI units. From ® Coulter Electronics Limited, Coldharbour Lane, Harpenden, Herts.

Parameter	Unit(s)	Range	Mean
**WBC**	10^9/L	6,4 - 26,2	15
**SED**	rate %	1 - 2	1,5
**HGB**	g / dl	10,8 - 17,5	14,4
	mmol / L	6,7 - 10,9	9
**HCT**	%	0,35 - 0,51	0,45
**PLT**	10^9 / L	190 - 1000	800
**RBC**	10^12 / L	7 - 10	8,6
**MCV**	fl	7 - 65	61
**MCH**	pg	15,0 - 22,0	17
	nmol / L	9,3 - 13,7	10,6
**MCHC**	%	30,0 - 35,0	32
	mmol / L	18,6 - 21,7	19,9
**NEUT**	%	12 - 46	30
**LYMPH**	%	53 - 83	67
**MONO**	%	1 - 7	5
**EOS**	%	0 - 3,5	2,1

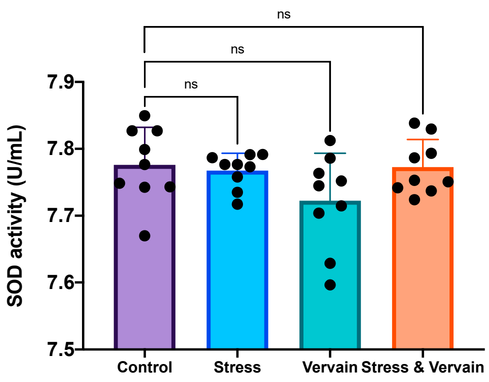

Figure S2. Superoxide dismutase, SOD, activity (Randox®) in whole blood of rats. No significant differences in SOD activity were registered due to stress or vervain administration. Median marked with horizontal bar. ns - non-significant p value.
